# Skin blood flow and skin temperature 24 h after induced muscle damage

**DOI:** 10.14814/phy2.70410

**Published:** 2025-06-10

**Authors:** Clara Carrión‐González, Andrea Martínez‐Santarrufina, Carlos Libio Escrivá‐Estellés, Lukas Verderber, Inmaculada Aparicio‐Aparicio, Jose Ignacio Priego‐Quesada

**Affiliations:** ^1^ Research Group in Sports Biomechanics (GIBD), Department of Physical Education and Sports Universitat de València Valencia Spain; ^2^ Motor Control, Cognition and Neurophysiology, Institute of Human Movement Science & Health Chemnitz University of Technology Chemnitz Germany; ^3^ Research Group in Medical Physics (GIFIME), Department of Physiology Universitat de València Valencia Spain

**Keywords:** delayed onset muscle soreness, infrared thermography, laser doppler, perfusion, recovery

## Abstract

Infrared thermography is a noninvasive tool to monitor muscle damage, though its effectiveness is debated, as some studies report no skin temperature changes 24–48 h post‐damage. These findings are often attributed to skin blood flow, but there is a lack of studies assessing this outcome. This study aimed to assess baseline skin temperature and skin blood flow responses 24 h after an induced quadriceps damage protocol and to establish a possible relationship between both variables. The thigh skin temperature and skin blood flow, pain perception, serum creatine kinase, and height of countermovement jump were measured in 15 physically active adults over 4 days, on two consecutive days per week. The induced muscle damage protocol, based on 100 drop jumps, was performed on the third day. Higher serum creatine kinase and pain perception (*p* < 0.01, ES > 1.20) were found 24 h after muscle damage induction. Thigh skin temperature and skin blood flow were unaffected by the muscle damage protocol, and a moderate correlation was observed between both outcomes (*r* = 0.59). We conclude that muscle damage does not alter skin blood flow 24 h after the induction, and therefore, this also had no consequences on skin temperature.

## 1 INTRODUCTION

Delayed onset muscle soreness (DOMS) is a phenomenon that arises after performing exercise that induces muscle damage, often related to unusual physical activity or eccentric contractions, and which normally appears between 24 h (Byrne et al., 2004) and 48 h after the activity (da Silva et al., 2021; de Carvalho et al., 2021). Muscle damage and DOMS may include mechanical strains and disruption to muscle structures, and biochemical reactions and inflammatory processes that can result in transient strength loss, reduced joint range of motion, and lower sports performance (Doménech‐García et al., 2023). For this reason, the tools to analyze muscle damage and DOMS are vital for monitoring internal training load and reducing injury risk to the athlete (Halson, 2014). Muscle biopsies and magnetic resonance imaging are direct methods of assessing muscle damage (Clarkson & Hubal, 2002), but are invasive, expensive, and difficult to access (Meola et al., 2012; Schvartzman et al., 2016). Indirect methods include the quantification of muscle proteins or other biomarkers (Rojas‐Valverde et al., 2021), focusing on the increase in serum creatine kinase (CK) as one of the most often used biomarkers of musculoskeletal disorders (Banfi et al., 2012; Brancaccio et al., 2010), even though this presents wide variability between subjects (Clarkson et al., 1992). Numerical scales, questionnaires, and visual analog scales (VAS) are also used for the evaluation of DOMS (Byrne et al., 2004; Rojas‐Valverde et al., 2021), with the limitation of associated subjectivity (Jensen et al., 1989). A correlation has also been shown between exercise‐induced damage and a decrease in countermovement jump (CMJ) height (García‐López et al., 2006; Pérez‐Guarner et al., 2019; Rodrigues Júnior et al., 2021), but this also presents limitations in athletes accustomed to plyometric training (Stojanović et al., 2017). Since all the methods have associated limitations, research continues to investigate the possibility of complementing them with new tools.

Infrared thermography (IRT) is now being used for analyzing muscle damage and DOMS because it is an imaging, noncontact (not interfering with thermoregulation) and noninvasive method used for measuring skin temperature (T_sk_) (dos Santos et al., 2022). Thermoregulation is a thermal control function governed by the hypothalamus, responsible for maintaining constant core temperature despite exposure to different activity or environmental conditions (Cramer & Jay, 2016; Morrison, 2016). An increase in inflammatory activity could lead to an increase in intramuscular temperature, which could be associated with increased blood perfusion to dissipate heat (Charkoudian, 2010; González‐Alonso, 2012). In this way, it was suggested that the rise in intramuscular temperature due to the inflammatory process could also be reflected in an increase in T_sk_ and interpreted by IRT (de Andrade Fernandes, 2017; Hildebrandt et al., 2010).

However, the relationship between T_sk_ and muscle damage is still unclear (da Silva et al., 2018; Rojas‐Valverde et al., 2021). Several studies found no evidence of T_sk_ responses at 24 or 48 h after a damage protocol (Pérez‐Guarner et al., 2019; Priego‐Quesada et al., 2020), and others did not find any correlations between T_sk_ and damage indicators such as CK and DOMS appearance (da Silva et al., 2021; de Carvalho et al., 2021; dos Santos et al., 2022). Research points to the hypothesis that the nonresponse of T_sk_ may be due to a higher peripheral vasoconstriction caused by DOMS and muscle damage (Zambolin et al., 2022). The sensitization of nociceptive and mechanosensitive afferents following muscle damage may reduce skin blood flow (BF_sk_) by increasing sympathetic activity and vascular resistance (Zambolin et al., 2022). In particular, reduced resting femoral blood flow and leg vascular conductance have been reported when both nociceptive and mechanoreceptor pathways are activated, suggesting that vasoconstriction may be mediated by autonomic mechanisms (Zambolin et al., 2022). Therefore, to understand the complexity of thermoregulatory processes, the use of IRT should be combined with techniques that provide BF_sk_ measurements (Johnson & Kellogg, 2010; Priego‐Quesada et al., 2020). However, to the authors' knowledge, none of the previous investigations that have used IRT to detect muscle damage or DOMS were accompanied by BF_sk_ measurement.

The objective of our study was to assess T_sk_ and BF_sk_ responses 24 h after an induced muscle damage protocol and to establish a possible relationship between both variables, muscle damage outcomes (DOMS, CK, and jump height) and a confounding variable (body fat composition). We hypothesized, based on previous research (Ferreira‐Júnior et al., 2021; Pérez‐Guarner et al., 2019; Priego‐Quesada et al., 2020), that T_sk_ would not be altered 24 h after a muscle damage induction protocol, and the nonresponse of T_sk_ would be explained by the non‐alteration or decrease of BF_sk_.

## 2 MATERIALS AND METHODS

### 2.1 Participants

A sample size of 14 participants for a repeated measures ANOVA was calculated considering an effect size (Cohen's f) of 0.3 (moderate effect size), an *α* of 5%, a power of 90%, 4 days of repeated measurements, and a correlation among repeated measures of 0.7 (G*Power 3 software, University of Düsseldorf, Düsseldorf, Germany) for mean T_sk_ outcome. Effect size and correlation estimations were obtained after the analysis of the first five participants. Then, 15 volunteers (11 males and 4 females) participated in the study (24 ± 3 years old, 77.5 ± 19.0 kg, 175 ± 10 cm, and 18 ± 8% of body fat). The inclusion criteria were to be physically active, performing at least two sessions of physical activity per week, and be between 18 and 40 years old. Exclusion criteria were to suffer from lower limb neuromuscular injury at least 4 months before the measurements, being accustomed to performing eccentric work, smoking, and any pathological or metabolic disease, especially circulatory disorders. A previous online questionnaire was disseminated to social media and by emails to the Bachelor of Physical Activity and Sports Sciences students of the University of Valencia, to obtain the necessary data to recruit participants following the criteria mentioned. The Ethics Committee of Research in Humans of the University of Valencia approved this research (application reference 2023‐FIS‐3184013) which was in agreement with the Declaration of Helsinki, and all participants signed a consent form agreeing to participate.

Several instructions were provided aimed at reducing T_sk_ and BF_sk_ variability. Participants could not be enrolled in any systematic physical training during the event, and they were instructed to not use anti‐inflammatory substances, physiotherapy treatments, or sunbathe on the day before measurements were taken; to avoid smoking, alcohol consumption, or stimulant beverages 12 h prior to the test; to not eat heavy meals, ensuring that the last one was consumed at least 2 h before the study; to abstain from applying creams on the thigh and guarantee a minimum of 7 h of sleep the night before the measurements (Priego‐Quesada et al., 2022). The participants confirmed compliance with these instructions verbally on each day of measurement.

### 2.2 Study design

Data collections were undertaken over 4 days in two consecutive weeks: two consecutive days per week, with an interval of 24 h between day one and two, and day three and four. On the first day, the participants were submitted to an anthropometrical assessment with a stadiometer (height) and a bioelectrical impedance system to measure fat percentage (Tanita BC‐545M, Tanita Corp., Japan). Days 1 and 2 were used for control measurements (Control day 1 and Control day 2). On the third day of the study (Pre‐muscle damage), a Drop Jump (DJ) protocol to induce muscle damage in the quadriceps was applied. Day 4 (post‐muscle damage) was similar to days 1 and 2, aimed at evaluating the responses in the different outcomes 24 h after the DJ protocol.

An exercise‐induced damage protocol was performed only on pre‐muscle damage day at the end of the session. It consisted of a validated protocol of DJ to induce muscle damage in the knee extensors, including 5 sets of 20 DJs from a 0.6 m box, with a rest of 10 s between jumps and 2 min between sets (Hohenauer et al., 2020; Miyama & Nosaka, 2004). The DJ technique was verbally explained and physically demonstrated: (1) initial standing position on the box, (2) drop, (3) take‐off fast when landing, and jumping up as high as possible. The exercise was modified to emphasize bending the knees when landing to encourage damage to the rectus femoris through the eccentric component.

**FIGURE 1:**
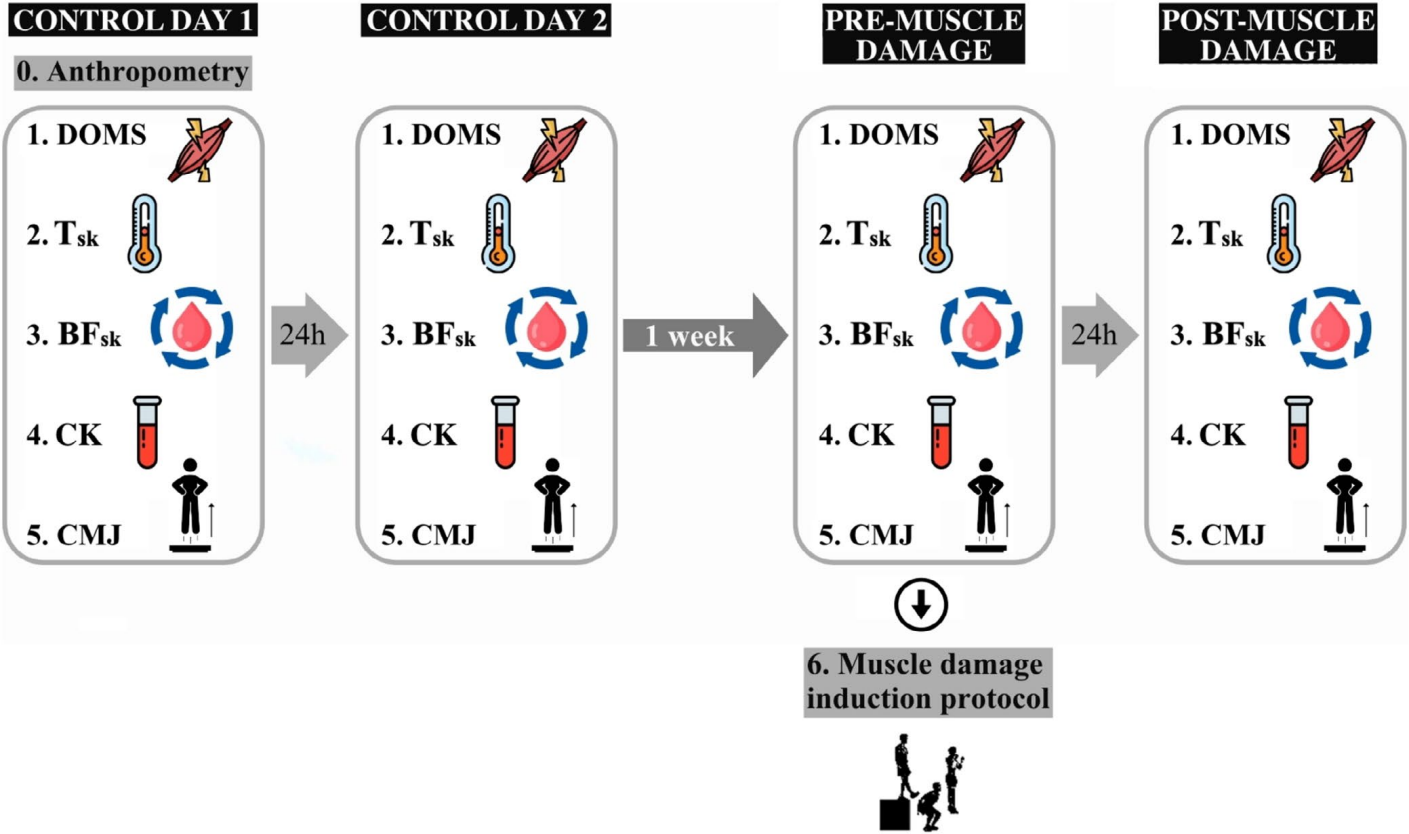
Study design for assessing anthropometry, delayed onset muscle soreness (DOMS), skin temperature (T_sk_), peripheral blood flow (BF_sk_), serum creatine kinase (CK), countermovement jump (CMJ), and muscle damage induction protocol over four testing days.

Environmental conditions were as follows: room temperature 22.7 ± 1.0°C and relative humidity of 38 ± 8% (without significant differences between sessions). All variables were measured in each session, and measurements were undertaken in the same order and manner for all participants. Also, each procedure was always recorded by the same researcher (Figure 1).

### 2.3 Procedures

#### 2.3.1 Pain perception

At the beginning of each session, a VAS of 150 mm (Price et al., 1983) was applied to assess DOMS intensity of quadriceps femoris. The VAS was explained to the participants, where the left side of the scale was labeled as “no pain” and the right side as “worst pain possible”. The participants were asked to put a mark on the line, after palpation of the area to be measured, in response to the question “How sore are your thighs today?”. The pain perception score was obtained by measuring, with a ruler, where their mark was; 0 marked the beginning of the scale on the left, and 15 marked the end of the line on the right.

#### 2.3.2 Infrared thermography

T_sk_ was quantified using an IRT camera (FLIR model E60, Flir Systems Inc., Wilsonville, Oregon, USA) with a resolution of 320 × 240 pixels, an instantaneous field of view (IFOV) of 1.36 mrad, with noise‐equivalent temperature difference (NETD) < 0.05°C and measurement uncertainty of ±2°C. The camera was turned on at least 10 min before the evaluations to allow stabilization of the electronic components. It was positioned 50 cm away from the participant and with the lens aligned perpendicular to the body region of interest (ROI). A 10‐min room thermal adaptation took place with the anterior thigh uncovered due to clothing, and all images were recorded with participants seated. An emissivity factor of 0.98 was set, and all recommended procedures for sports thermographic imaging regarding control of environment temperature, humidity, and participant preparation were followed to minimize factors that could influence the measurement (Moreira et al., 2017).

**FIGURE 2:**
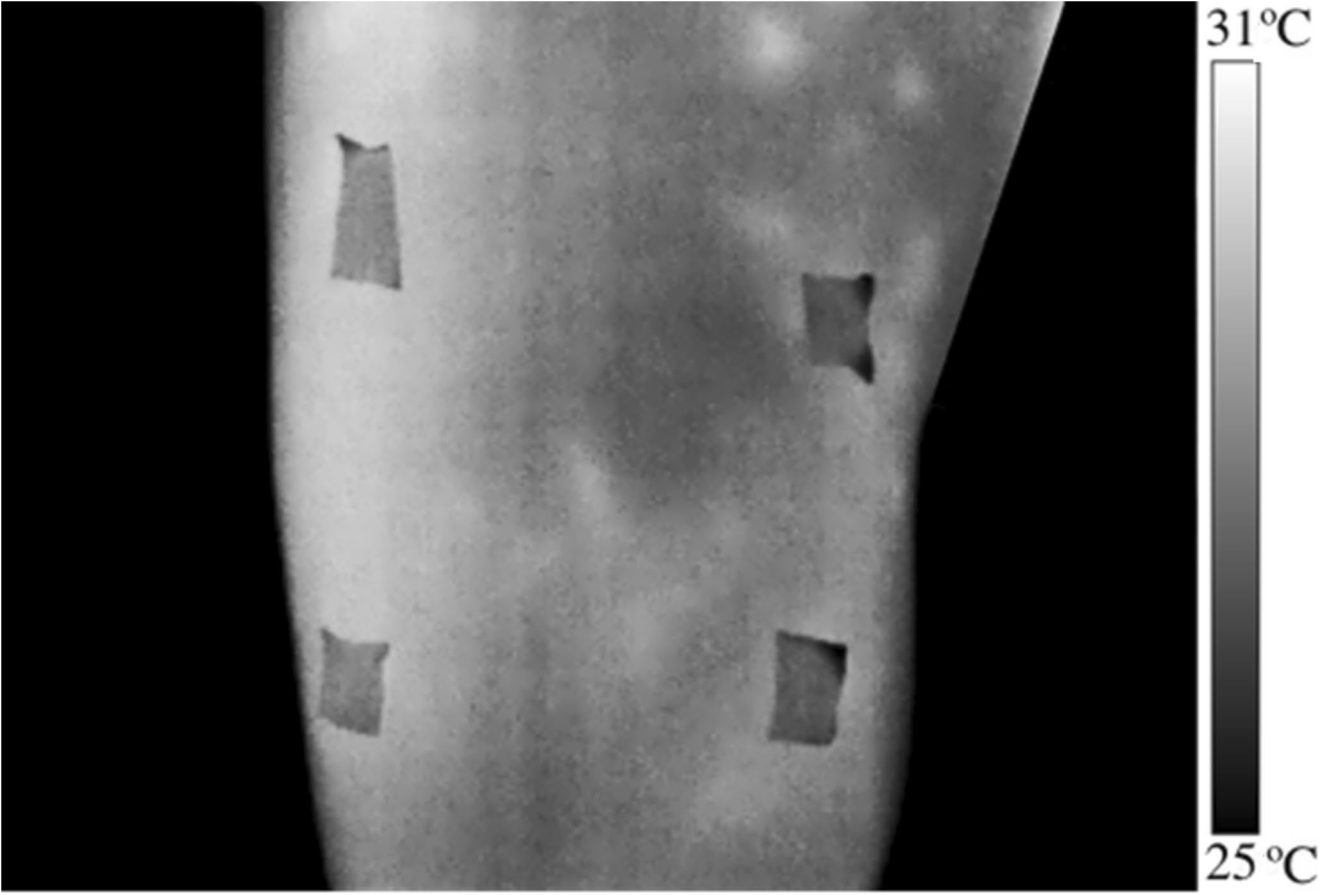
Definition of the ROI in anterior thigh for analyzing skin temperature.

ROI assessment site was located and marked on the right leg, in the rectus femoris: 20 cm proximal to the base of the patella, with a size of 6 × 7 cm (Figure 2). These parameters were decided on to establish a standard measurement for all participants and to delimit the same BF_sk_ scanning area. We decided to focus on only one lower limb, as T_sk_ asymmetries do not typically appear following bilateral muscle damage induction (Verderber et al., 2024). T_sk_ was determined considering the mean, maximum, minimum, and standard deviation of the ROI with an emissivity of 0.98, using commercial software (Thermacam Researcher Pro 2.10 software, FLIR, Wilsonville, OR, USA).

#### 2.3.3 Laser doppler

A laser Doppler scan (moorLDI2, Moor Instruments Ltd., Devon, United Kingdom) was used to quantify the BF_sk_. Due to the lack of literature on the perfusion imaging of post muscle damage in the context of sports science, all the parameters established for the measurement were decided upon by the members of the research team, following pilot studies and taking into account the recommendations of the general guideline for laser Doppler (Fullerton et al., 2002). Scanning was performed in a seated position with a laser‐thigh distance of 40 cm from all participants, each measurement being adjusted for each participant, as the color of this tissue and the level of light captured by the detector depend on the distance (Fullerton et al., 2002). Setting the image size to a small area covering the ROI tissue minimizes the total imaging time, thus maximizing image integrity. Therefore, the scan resolution was 120 × 140 pixels, obtaining a speed scan of 10 ms/pixel, resulting in a scanning time of 3 min and 36 s.

**FIGURE 3:**
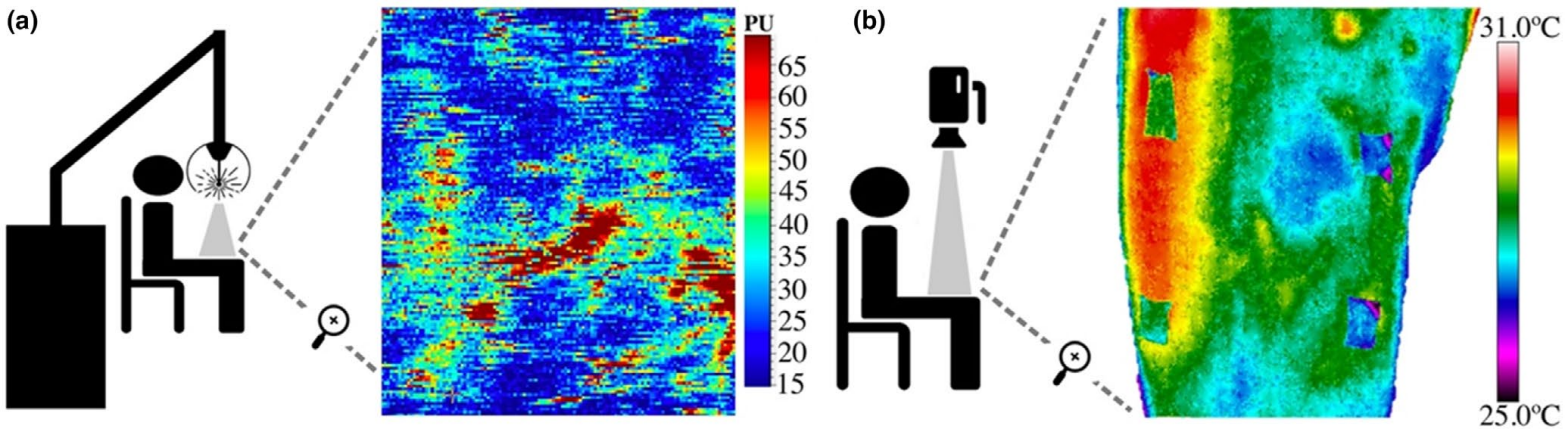
Laser Doppler (a) and IRT camera (b) placements and images.

The assessment area was the same as T_sk_ (Figure 3). Participants were instructed to remain as still as possible during the measurement to avoid distorting the images: sitting in a comfortable position, looking straight ahead, and not talking. The manufacturer's software (MoorLDI V6.2 software, Moor Instruments Ltd., Devon, UK) was used to obtain the mean, maximum, minimum, and standard deviation of the BF_sk_ in arbitrary perfusion units (PU). The vascularization data of the ROI had a range of 0–5000 PU.

#### 2.3.4 Serum creatine kinase

Blood was collected after laser Doppler to obtain CK by SimplexTAS 101 Analyzer and cartridges SimplexTAS 101 CK (Sysmex Europa, Bornbarch, Germany), with a measuring range of 10–1200 IU/L. The volume of blood required was 20 μL, taken from the finger by capillary puncture using a tip tub, later stored in a reagent cartridge and introduced into the automatic analyzer.

The SimplexTAS 101 Heating Block (Sysmex Europa, Bornbarch, Germany) was used as an incubator to warm up the reagent cartridges each session before testing. While not being tested, cartridges were stored in a cooler at 5°C. All procedures were performed under relevant protocols for the handling and disposal of biological materials, according to the manufacturer's instructions for the equipment and reagents used.

#### 2.3.5 Jump performance

The CMJ trial took place after blood collection to evaluate the lower limbs' power, variations being observed in jump height as an indicator of fatigue related to muscle damage (García‐López et al., 2006). The participants were instructed to jump as high as possible starting from a standing position with the hands on the hips, squatting down quickly, reaching 90° of knee flexion and then extending it in one continuous and fastest possible movement to jump and land on their toes.

For data collection, participants performed a warm‐up consisting of 10 squat exercise repetitions. Three CMJs were performed using a Chronojump platform (model DIN‐A3, Chronojump Bosco‐System, Barcelona, Spain) with 20 s of rest between the repetitions (Brocherie et al., 2014). The mean of the jumps was used for analyzing jump height (Pérez‐Guarner et al., 2019). The measurements were recorded and analyzed with the software Chronojump (version 1.4.7.0., Chronojump‐Bosco system, Barcelona, Spain).

### 2.4 Statistical analysis

The statistical analysis was conducted using RStudio (version 2024.04.1), primarily employing the “rstatix” (version 0.7.2), “ggplot2” (version 3.5.0), “effectsize” (version 0.8.6), “nlme” (version 3.1–166), and “ggcorrplot” (version 0.1.4.1) packages. The significance threshold was set at 0.05. The normality of the variables was assessed using the Shapiro–Wilk test, observing that, except for mean, maximum, and minimum T_sk_ and jump height (*p* > 0.05), the remaining variables did not follow a normal distribution. For nonparametric variables, Friedman tests were performed to assess the effect of the week factor (Control vs. Experimental weeks) and the session factor (Control day 1 vs. Control day 2, or Pre vs. Post‐Muscle damage) with Wilcoxon pairwise comparisons and Bonferroni adjustment. For parametric variables, a repeated measures ANOVA was employed with two main factors (session and week) and the interaction between them, and with Bonferroni‐adjusted Student's *t*‐test for pairwise comparisons. For significant differences observed, 95% confidence intervals (CI95%) were presented and Hedge's effect sizes (ESg) were computed with paired correction and classified as small (ESg 0.2–0.5), moderate (Esg 0.5–0.8), or large (ESg >0.8) (Cohen, 1988; Hedges, 1981).

Additionally, a Pearson bivariate correlation study was conducted for mean T_sk_, mean BF_sk_, CK, pain perception, jump height, and fat percentage, using the data of the four sessions. Significant correlations (*p* < 0.05) were classified as weak (0.2 < |*r*| < 0.5), moderate (0.5 ≤ |*r*| < 0.8), or strong (|*r*| ≥ 0.8) (O'Rourke et al., 2005). Finally, to explore multiple relationships explaining mean T_sk_ and mean skin blood flow, two multivariate mixed regression models were performed: one to explain mean T_sk_ based on mean BF_sk_, CK, pain perception, jump height, and fat percentage, and another to explain mean BF_sk_ using the same variables, substituting mean BF_sk_ for mean T_sk_. The participant was used as a randomized factor adjusted for the intercept. Final models were then adjusted to retain only variables yielding *p* values <0.05. The assumption of normality for the residuals to the fitted values and convergence were confirmed for each mixed model obtained. The models were provided with the marginal *R* ^2^ of the fixed effects (without random effects) and the conditional *R* ^2^ that includes the fixed and random effects.

## 3 RESULTS

### 3.1 Effect of induction damage protocol on damage parameters

**FIGURE 4:**
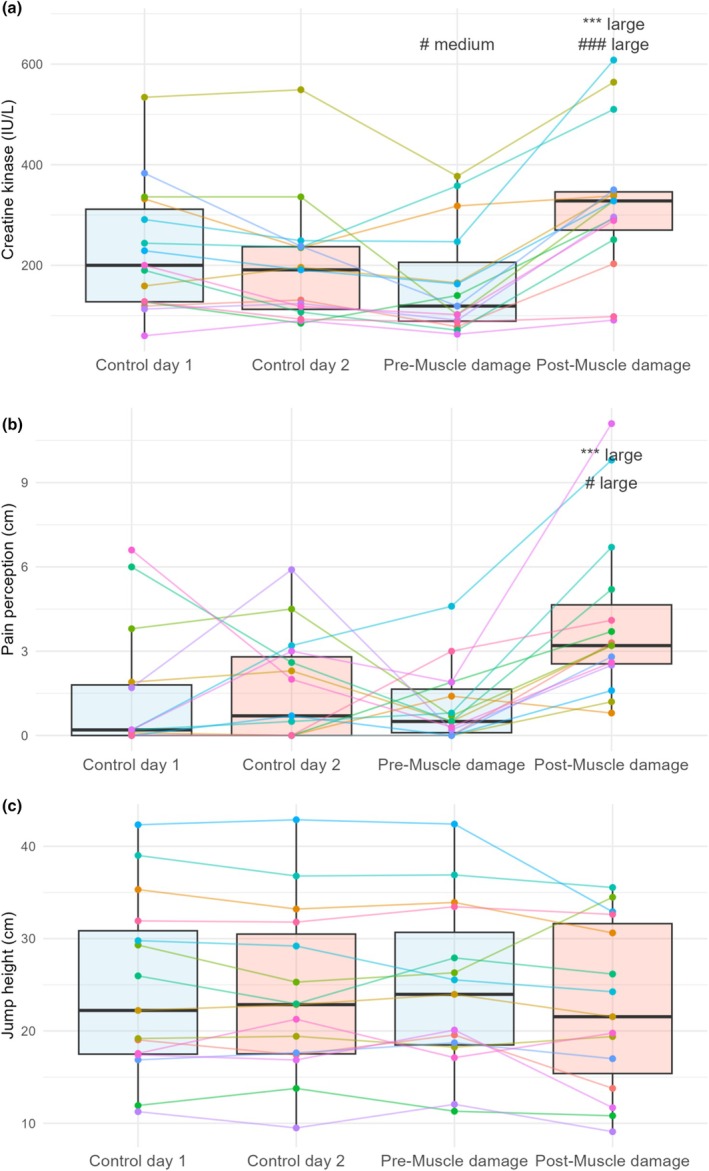
Box plots (median, interquartile range, and whiskers to 1.5× interquartile range) of the differences between days in parameters related to muscle damage ([a] creatine kinase, [b] pain perception, and [c] jump height). Points and lines show the individual responses. Differences are indicated using symbols (***p< 0.001 diff with Pre‐Muscle damage; and ^#^ *p* < 0.05 ^###^ *p* < 0.001 diff with the same day of the Control week) and the magnitude of the effect size.

There was a significant effect of the factors week and session on CK (Figure 4a; *p* < 0.001 for both factors) and VAS (Figure 4b; *p* < 0.001 for week and *p* < 0.01 for session). However, jump height was not altered over the different days (Figure 4c; *p* = 0.21 for week, *p* = 0.06 for session, and *p* = 0.32 for the interaction between week and session).

CK at Pre‐Muscle damage was lower than at Control day 1 (*p* = 0.02, ES = 0.53, and 95% CI of the difference [14.0, 127.5 IU/L]), and Post‐Muscle damage was higher than Control day 2 (*p* < 0.001, ES = 0.92, and 95% CI [69.0, 180.0 IU/L]) and Pre‐Muscle damage (*p* < 0.001, ES = 1.21, and 95% CI [69.0, 180.0 IU/L]).

Pain perception was higher Post‐Muscle damage than Control day 2 (*p* = 0.01, ES = 0.96, and 95% CI [0.9, 4.4 cm]) and Pre‐Muscle damage (*p* < 0.001, ES = 1.30, and 95% CI [1.8, 4.2 cm]).

### 3.2 Effect of induction damage protocol on skin blood flow and skin temperature response

**FIGURE 5:**
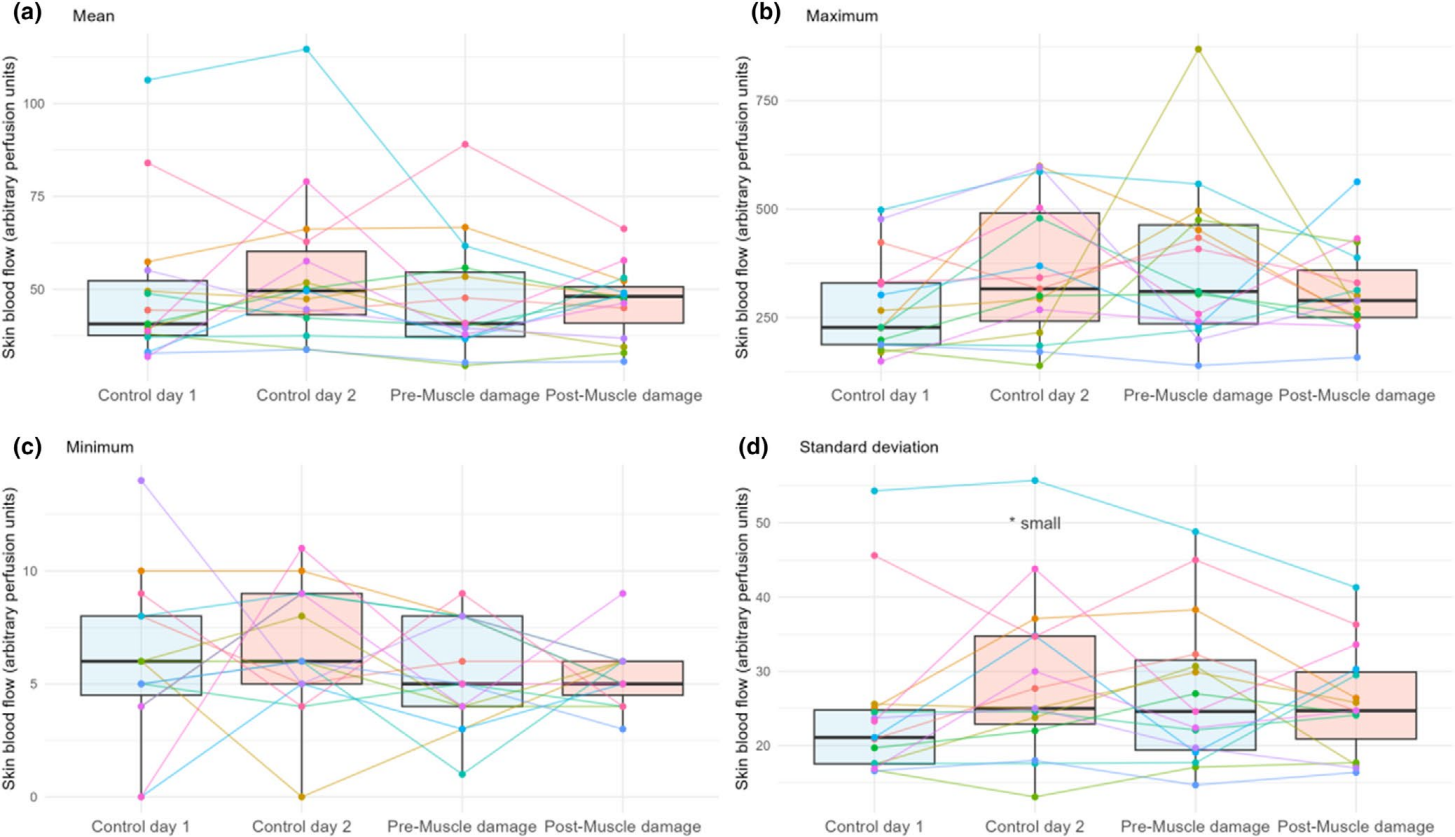
Box plots (median, interquartile range, and whiskers to 1.5× interquartile range) of the differences between days in the different parameters of skin blood flow ([a] mean, [b] maximum, [c] minimum, and [d] standard deviation). Points and lines show the individual responses. Differences are indicated using symbols (*p < 0.05 diff with Control day 1) and the magnitude of the effect size.

BF_sk_ was not altered 24 h after muscle damage induction protocol for mean (Figure 5a; *p* = 0.44 for week, *p* = 0.44 for session), maximum (Figure 5b; *p* = 0.07 for week, *p* = 0.07 for session), and minimum (Figure 5c; *p* = 0.21 for week, *p* = 0.41 for session). SD of BF_sk_ had an effect of session (Figure 5d; *p* = 0.44 for week, *p* = 0.03 for session), showing lower values at Control day 1 than Control day 2 but with a small effect size (*p* = 0.03, ES = 0.38, and 95% CI [0.4, 10.0 PU]).

**FIGURE 6:**
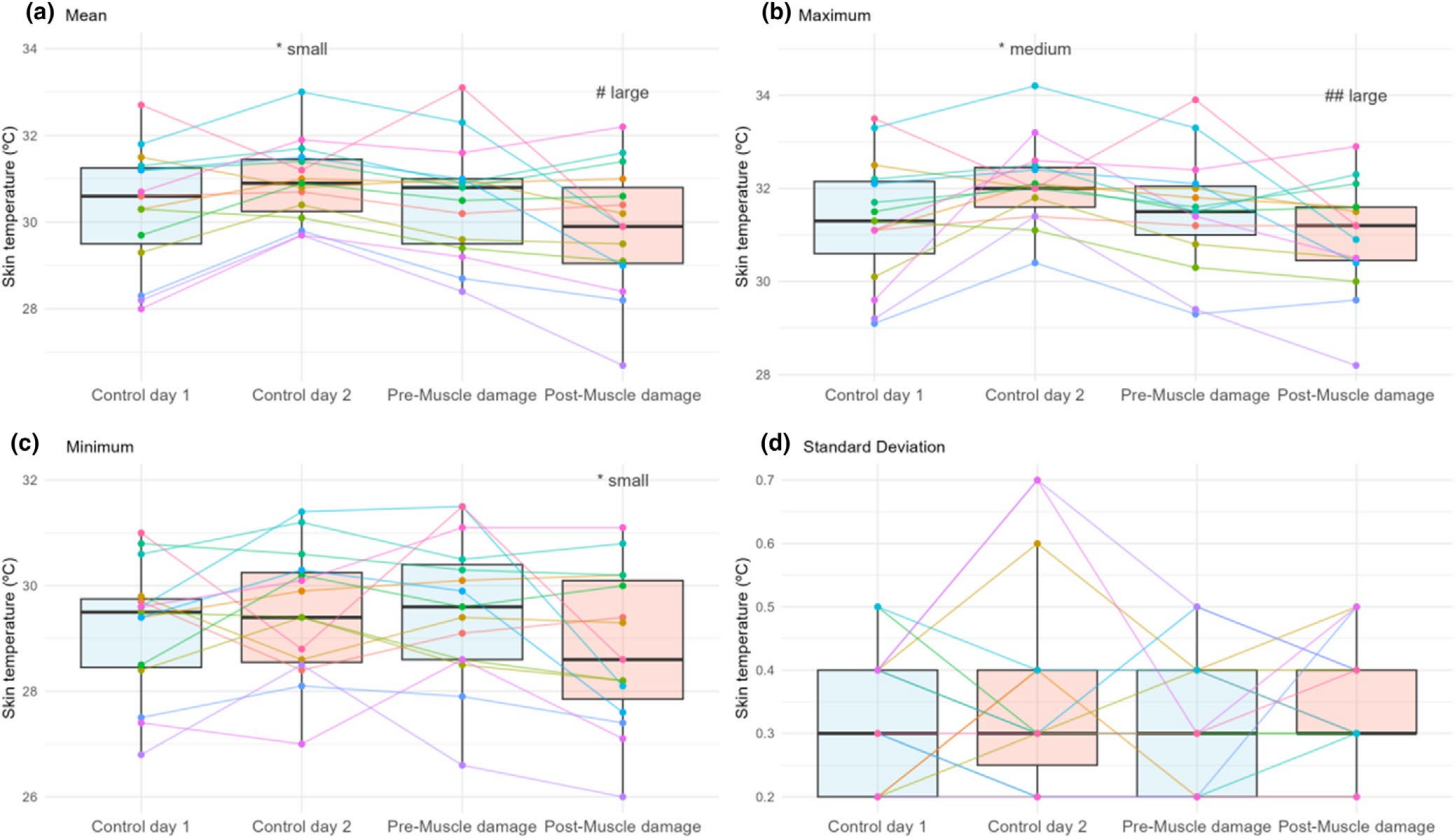
Box plots (median, interquartile range, and whiskers to 1.5× interquartile range) of the differences between days in the different parameters of skin temperature (a: mean, b: maximum, c: minimum, and d: standard deviation). Points and lines show the individual responses. Differences are indicated using symbols (*p < 0.05 diff with Control day 1 or Pre‐Muscle damage; and ^#^ *p* < 0.05 ^##^ *p* < 0.01 diff with the same day of the Control week) and the magnitude of the effect size.

There was a significant main effect of week (mean *p* = 0.01; maximum *p* = 0.02) and an interaction of week and session (mean *p* < 0.01; maximum *p* < 0.01) for mean and maximum T_sk_. For minimum T_sk_, the only significant result was the interaction between week and session (*p* = 0.04). Mean T_sk_ (Figure 6a) and maximum T_sk_ (Figure 6b) were higher on Control day 2 than on Control day 1 (mean: *p* = 0.03, ES = 0.48, and 95% CI [0.1, 1.1°C]; maximum: *p* = 0.03, ES = 0.67, and 95% CI [0.1, 1.5°C]) and on the Post‐Muscle damage (mean: *p* < 0.01, ES = 0.48, and 95% CI [0.4, 1.7°C]; maximum: *p* < 0.01, ES = 1.04, and 95% CI [0.5, 1.8°C]). Minimum T_sk_ was lower at the Post‐Muscle damage than at the Pre‐Muscle damage (*p* = 0.04, ES = 0.49, and 95% CI [0.1, 1.4°C]).

### 3.3 Relationship between variables

**FIGURE 7:**
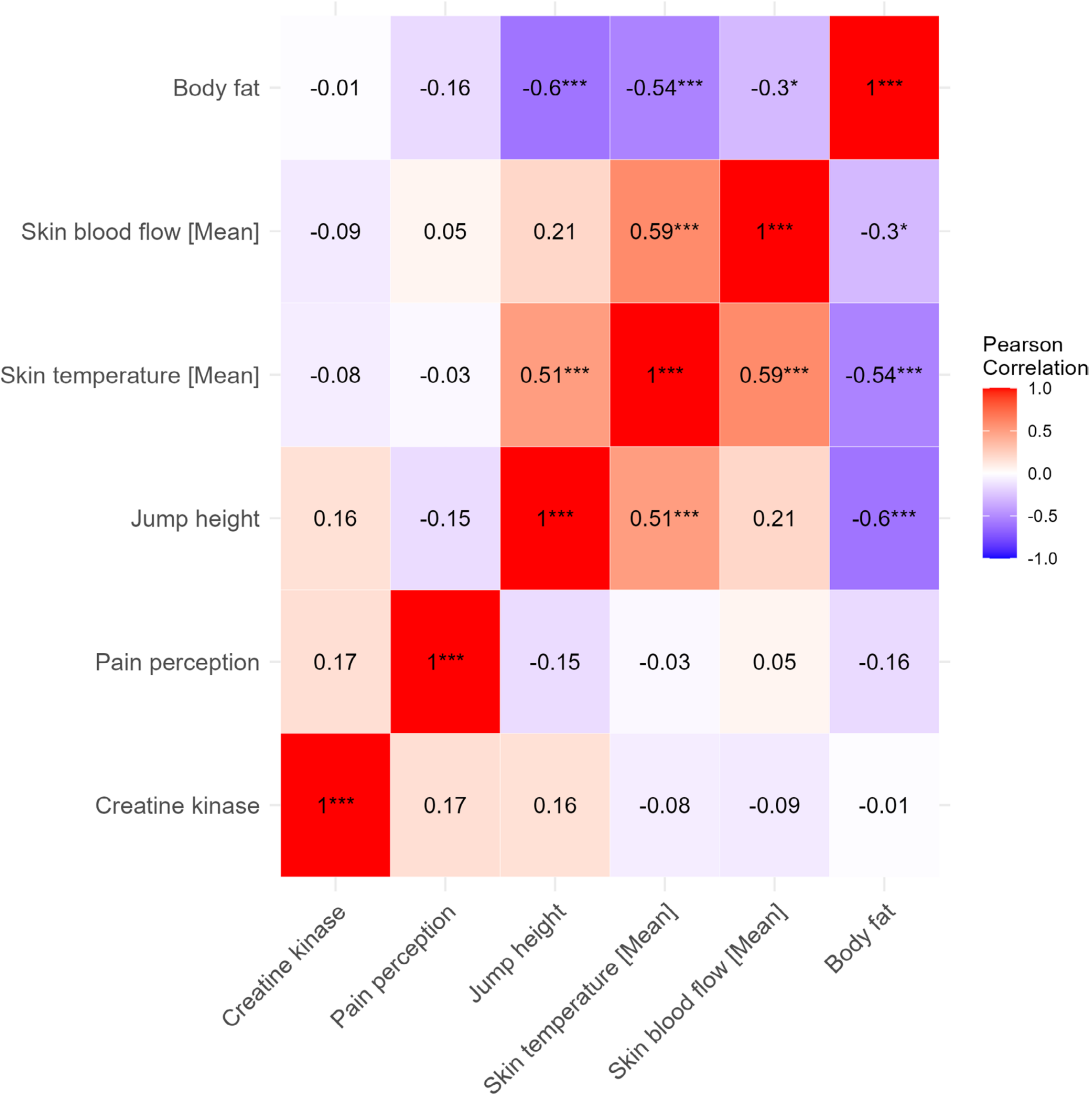
Bivariate correlation Pearson analysis of the variables. Significant correlations were indicated by symbols (*p < 0.05; ***p < 0.001).

**FIGURE 8:**
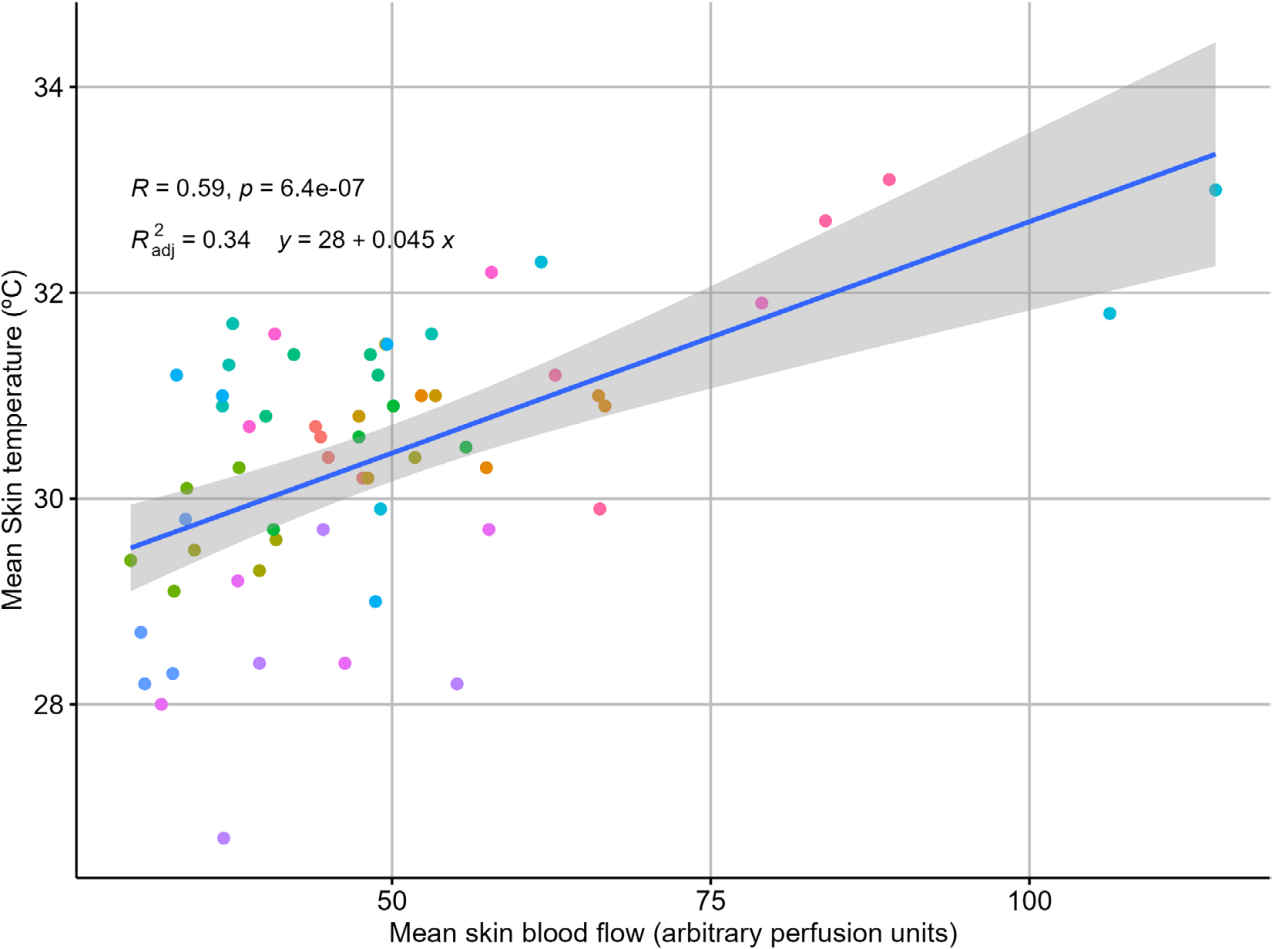
Relationship between mean skin blood flow (perfusion units) and mean skin temperature (°C) across participants. Each point represents an individual data pair, with colors indicating different participants. A linear regression line is shown with its corresponding 95% confidence interval (shaded area). The Pearson correlation coefficient and the regression equation are displayed on the graph.

Bivariate correlation analysis (Figure 7) showed that mean T_sk_ was moderately and directly related to jump height (*r* = 0.51 and *p* < 0.001) and mean BF_sk_ (*r* = 0.59 and *p* < 0.001), and moderately inversely associated with body fat percentage (*r* = −0.54 and *p* < 0.001). Body fat percentage was also inversely associated with jump height (*r* = −0.60 and *p* < 0.001) and mean BF_sk_ (*r* = −0.30 and *p* = 0.02). CK and pain perception were unrelated to any other parameters (*p* > 0.05). Figure 8 shows the scatterplot of the correlation between mean T_sk_ and mean BF_sk_.

Mixed regression models (Table 1) showed that 46% of the variance of fixed effects of the mean T_sk_ was explained by jump height and mean BF_sk_, while mean BF_sk_ was only explained by mean T_sk_ with 33% of the variance.

**TABLE 1:** Mixed regression model results for mean skin temperature and mean skin blood flow.

Predictors	Skin temperature			Skin blood flow		
	Estimates	CI	*p*	Estimates	CI	*p*
(Intercept)	27.29	26.12–28.47	**<0.001**	−180.62	−274.78 – −86.46	**<0.001**
Jump height (cm)	0.06	0.02–0.10	**0.005**			
Skin blood Flow (PU)	0.03	0.02–0.05	**<0.001**			
Skin temperature (°C)				7.56	4.47–10.65	**<0.001**
Random effects						
σ^2^	0.48			104.60		
τ_00_	0.45 _PARTICIPANT_			96.09 _PARTICIPANT_		
ICC	0.48			0.48		
N	15 _PARTICIPANT_			15 _PARTICIPANT_		
Observations	60			60		
Marginal *R* ^2^/Conditional *R* ^2^	0.455/0.719			0.327/0.649		

*Note*: Significant values (*p* < 0.05) are highlighted by bold letters.

## 4 DISCUSSION

This study aimed to analyze T_sk_ and BF_sk_ responses after an induced muscle damage protocol and whether there is a possible relationship between both variables and with DOMS, CK, jump height, and body fat composition. Muscle damage was indirectly measured by pain perception, CK, and changes in CMJ height. In this sense, higher values of pain perception and CK were observed 24 h after the muscle damage induction protocol. Our main findings indicate that although some statistically significant differences were observed in T_sk_ and BF_sk_ across sessions, there was no increase in these variables 24 h after muscle damage when compared to baseline or to the control week. This supports the notion that neither T_sk_ nor BF_sk_ shows a consistent elevation as a result of the induced muscle damage. As far as the authors know, this work is the first to assess the effect of muscle damage on both T_sk_ and BF_sk_ responses.

The increase of CK and pain perception are markers commonly used by previous studies to verify the presence of muscle damage and DOMS (Baird et al., 2012; da Silva et al., 2021; García‐López et al., 2006; Howatson et al., 2009). The rise of CK and pain perception in our study confirmed that muscle damage and DOMS result from the eccentric phase of the DJs, in agreement with previous studies using this protocol (Hohenauer et al., 2020; Miyama & Nosaka, 2004). However, the decrease in CMJ height when there is muscle damage which is reported in the literature (García‐López et al., 2006; Pérez‐Guarner et al., 2019; Rodrigues Júnior et al., 2021) was not significant in this investigation. Miyama and Nosaka (2004) suggested that factors such as muscle power and the timing of muscular activation determine the jump performance rather than metabolic factors, which are more relevant for the magnitude of eccentric exercise‐induced muscle damage. Moreover, different studies have suggested a limited sensitivity of jump height to detect early neuromuscular fatigue or muscle damage than other mechanical variables (e.g., eccentric duration and force at zero velocity) as the neuromuscular strategy used during the jump may adapt to preserve jump height despite underlying fatigue (Gathercole, Sporer, et al., 2015; Gathercole, Stellingwerff, & Sporer, 2015). Another possible explanation is that peak in DOMS and CK may occur at 48 h post exercise (Tofas et al., 2008) when the effect in CMJ could be higher. This aligns with a previous study using the same muscle damage induction protocol, which also did not observe a CMJ reduction 24 h post‐exercise and reported greater DOMS at 48 than at 24 h post‐exercise (Da Silva et al., 2024). However, although DOMS and CK values may increase and CMJ performance may decline at 48 h post‐exercise, based on previous studies that assessed both 24 and 48 h (Verderber et al., 2024), no substantial differences in T_sk_ responses would be expected.

Although the muscle damage and DOMS occurred 24 h after our muscle damage induction protocol, baseline T_sk_ was not altered, similar to previous studies that did not find T_sk_ responses 24 h or 48 h after a half marathon (Pérez‐Guarner et al., 2019), marathon (Priego‐Quesada et al., 2020), triceps surae muscle damage induction protocol (da Silva et al., 2018), or after a leg press resistance training (Ferreira‐Júnior et al., 2021). However, other studies observed increases of T_sk_ after two consecutive soccer matches (de Andrade Fernandes, 2017), after a marathon in a hot environment (Rojas‐Valverde et al., 2021), or after an intense single leg exercise (Stewart et al., 2020). These controversial results could be the result of differences in training; for instance for inactive muscle groups in single exercises serving as heat sinks so facilitating the role of vascularity to transfer heat away from the thigh (Stewart et al., 2020). Also, different responses of BF_sk_ in the days after exercise, for example, from being exposed to hot environmental temperatures that prevent peripheral vasoconstriction, were speculated upon as an explanation for the presence of higher T_sk_ after a marathon (Rojas‐Valverde et al., 2021) compared with similar studies at lower environmental temperatures where no T_sk_ peaks were observed (Priego‐Quesada et al., 2020). We can affirm that the IRT application depends on the powerful thermoregulatory system of the skin. In this sense, some alterations of T_sk_ were observed, specially higher values at the Control day 2. Possible explanations for the varying responses of T_sk_ include the multiple factors that influence temperature: skin blood flow, hydration, environment, nutrition, fitness level, or accumulated subcutaneous body fat (Priego‐Quesada, 2017). Finally, a lower minimum T_sk_ was observed 24 h after muscle damage induction compared to the previous day. Although this may be related to changes in pain threshold and vasoconstriction state (da Silva et al., 2021), it is important to interpret this finding with caution, as the effect size was small, and no significant difference was found compared to the same day in the control week.

The literature on BF_sk_ measured after muscle damage is very limited. It has been shown that the intramuscular blood flow rate increased after 48 h of eccentric exercise (Selkow et al., 2015). However, BF_sk_ can be affected differently as it has been observed that arterial blood flow cannot be correlated with underlying intramuscular perfusion (Kuznetsova et al., 1998; Saumet et al., 1988). The absence of changes to BF_sk_ may be due to limited blood available to the skin as the body prioritizes blood flow to damaged muscles for repair. Known as “functional sympatholysis”, sympathetic vasoconstriction can keep the BF_sk_ low to ensure an adequate supply to muscles and other vital organs (Ichinose et al., 2019). Moreover, nociceptive stimulation following muscle damage may further contribute to autonomic vasoconstriction, limiting skin perfusion even in resting conditions, and potentially explaining the absence of consistent increases in T_sk_. Likewise, the literature states that eccentric exercise can change the regulation of arterial and intramuscular blood flow by satisfying the metabolic response, which can accumulate analgesic mediators contributing to DOMS and deterioration of muscle function (Souza‐Silva et al., 2018). This also explains variations in blood perfusion depending on the occurrence of muscle damage or not.

Previous research showed that changes in T_sk_ 48 h after exercise did not relate to DOMS (da Silva et al., 2021), pain level, perception of recovery, and fatigue perception (de Carvalho et al., 2021), and CK (dos Santos et al., 2022). According to the correlation obtained between T_sk_ and BF_sk_, the lack of changes in T_sk_ after muscle damage could be due to the compensatory phenomenon of BF_sk_ after inflammation, as we did not find an increased BF_sk_, even though we can speculate that there is a rise in muscle blood flow as an immediate response to exercise associated with the metabolic demand (Clifford, 2011; Selkow et al., 2015). For this reason, some researchers hypothesized that exercise without pain increases T_sk_, while exercise with pain and muscle damage may increase peripheral vasoconstriction, causing unchanged T_sk_ (Priego‐Quesada et al., 2020). Thus, the influence of peripheral vasoconstriction phenomena would nullify the detection of these temperature changes occurring in more internal regions. Although our results did not show that BF_sk_ decreased 24 h after muscle damage, BF_sk_ did not increase, explaining the lack of T_sk_ responses.

Similar to our study, other authors are now providing evidence of the correlation between T_sk_ and BF_sk_ (Murray et al., 2009; Schlager et al., 2010), although a gap remains in the context of physical activity and sport. One study found a poor relationship between T_sk_ and perfusion values in the assessment of Raynaud's phenomenon, measured at a room temperature of 23°C and 30°C in the dorsal of the hand (e.g., *r* = 0.49 for the right hand at 30°C), the tip of the middle finger (e.g., *r* = 0.38 left middle 30°C) and the “gradient” between these (e.g., *r* = 0.47 left hand 30°C) (Clark et al., 2003). However, most current research on Raynaud patients shows a good correlation (*r* = 0.87) (Schlager et al., 2010). Moreover, this level of correlation between laser Doppler imaging and IRT was also observed at baseline (*r* = 0.67) and maximum (*r* = 0.73) blood flow and T_sk_ in participants who underwent cold stimulus with cold water (Murray et al., 2009). The fact that some of these correlations are slightly higher than what we observed in the present study could be related to the variability of the sample in terms of blood flow, as these are individuals with scleroderma spectrum disorders.

Moreover, the T_sk_ mixed regression model showed a moderate interdependence relationship not only with BF_sk_ but also with jumping height. The results of an observational study of soccer players had already suggested the possibility of using IRT in combination with the CMJ test because of the strong and positive correlation observed between strength and T_sk_ asymmetries before (*R* ^*2*^ = 0.43) and after (*R* ^*2*^ = 0.42) a competitive period (Rodrigues Júnior et al., 2021). Thus, there would be a similarity between CMJ and IRT in the detection of fatigue caused by muscle damage and inflammatory processes. However, T_sk_ responses to a half marathon were not able to predict physiological stress markers in runners (Pérez‐Guarner et al., 2019), as T_sk_ was not altered 24 and 48 h after the competition, despite the decrease in jump performance among other altered physiological stress parameters. We believe that the direct relationship we have observed between jump height and T_sk_ shows a relationship between physical level and T_sk_, in agreement with previous studies where individuals with a higher physical level have higher T_sk_ due to the fact that they are more efficient at dissipating heat through their body (Abate et al., 2013; Formenti et al., 2013).

Our study has several limitations. Most of the participants were male (*n* = 15), and our sample age was young (24 ± 3 years old). Therefore, it was not possible to assess whether the results vary depending on sex and age. Although participants were instructed to avoid confounding factors (see Section 2.1) and reported their compliance, we did not record their diet, sleep, or physical activity, so we cannot be certain that all participants followed the instructions completely. Also, participants, with the exception of physical activity, were able to perform their normal daily activities, which varied from person to person. We used this approach in our study so that the results would be more applicable to the field context. However, no records of these activities were kept for comparing what participants did between visits, which can be considered a limitation. Moreover, another day of measurements 48 h after the muscle damage protocol would be desirable; it was not feasible due to logistical constraints and participant availability, as most of them were physically active individuals unwilling to refrain from exercise for more than two consecutive days. Finally, considering the relevant findings of previous studies that examined the effect of cumulative training loads on T_sk_ (Korman et al., 2021; Machado et al., 2023), we encourage future research to explore this topic further by incorporating the assessment of BF_sk_ alongside T_sk_.

## 5 CONCLUSIONS

Although 24 h after a muscle damage induction protocol of DJs resulted in higher CK and pain perception, T_sk_ and BF_sk_ were not altered, and the relationship observed between them suggested their physiological connection in this response. A possible explanation is that whereas muscle damage could result in higher muscle blood flow, this is not reflected in higher BF_sk_, and therefore T_sk_ is not affected.

## FUNDING INFORMATION

This work was supported by the Spanish Ministry of Science, Innovation and Universities through the State Research Agency (Agencia Estatal de Investigación) under project PID2023‐152125NA‐I00. Moreover, the acquisition of the equipment for the project was funded by the Universitat de València (INV_AE.26462467) and the Generalitat Valenciana (Prometeo/2021/064).

## CONFLICT OF INTEREST STATEMENT

The authors declare no conflicts of interest.

## ETHICS STATEMENT

This study was approved by the Ethics Committee of Research in Humans of the University of Valencia (application reference 2023‐FIS‐3184013).

## Data Availability

The dataset used for the analysis of this work is in a public repository (https://data.mendeley.com/datasets/v6yj8cpj9s/1; DOI: 10.17632/v6yj8cpj9s.1).
